# Survival of a male patient harboring CASK Arg27Ter mutation to adolescence

**DOI:** 10.1002/mgg3.1426

**Published:** 2020-07-21

**Authors:** Konark Mukherjee, Paras A. Patel, Deepa S. Rajan, Leslie E. W. LaConte, Sarika Srivastava

**Affiliations:** ^1^ Fralin Biomedical Research Institute at Virginia Tech Carilion Roanoke VA USA; ^2^ Children's Hospital of Pittsburgh of UPMC University of Pittsburgh Pittsburgh PA USA; ^3^ Department of Internal Medicine Virginia Tech Carilion School of Medicine Roanoke VA USA

**Keywords:** CASK, cerebellar hypoplasia, electroencephalogram, epileptic encephalopathy, microcephaly

## Abstract

**Background:**

*CASK* is an X‐linked gene in mammals and its deletion in males is incompatible with life. *CASK* heterozygous mutations in female patients associate with intellectual disability, microcephaly, pontocerebellar hypoplasia, and optic nerve hypoplasia, whereas *CASK* hemizygous mutations in males manifest as early infantile epileptic encephalopathy with a grim prognosis. Here, we report a rare case of survival of a male patient harboring a *CASK* null mutation to adolescent age.

**Methods:**

Trio whole exome sequencing analysis was performed from blood genomic DNA. Magnetic resonance imaging (MRI), magnetic resonance spectroscopy (MRS), and electroencephalogram (EEG) analyses were performed to determine anomalies in brain development, metabolite concentrations, and electrical activity, respectively.

**Results:**

Trio‐WES analysis identified a *de novo* c.79C>T (p.Arginine27Ter) mutation in *CASK* causing a premature translation termination at the very N‐terminus of the protein. The 17‐years, and 11‐month‐old male patient displayed profound intellectual disability, microcephaly, dysmorphism, ponto‐cerebellar hypoplasia, and intractable epilepsy. His systemic symptoms included overall reduced somatic growth, dysautonomia, ventilator and G tube dependence, and severe osteopenia. Brain MRI revealed a severe cerebellar and brain stem hypoplasia with progressive cerebral atrophy. EEG spectral analysis revealed a global functional defect with generalized background slowing and delta waves dominating even in the awake state.

**Conclusion:**

This case study is the first to report survival of a male patient carrying a *CASK* loss‐of‐function mutation to adolescence and highlights that improved palliative care could extend survival. Moreover, the genomic position encoding Arg27 in *CASK* may possess an increased susceptibility to mutations.

## Graphical abstract

Loss‐of‐function mutations in the X‐linked gene CASK are frequently lethal in males or produce epileptic encephalopathy with severe neurodevastation. Here, we first report a male patient with CASK R27* null mutation having a nearly complete loss of CASK function and surviving to late adolescence.

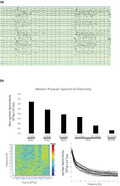

## 1 INTRODUCTION

Mutations in the X‐linked gene *CASK* (OMIM: 300172) are associated with intellectual disability, microcephaly and pontocerebellar hypoplasia (Burglen et al., 2012; Hayashi et al., 2012; LaConte et al., 2018, 2019; Moog et al., 2011; Najm et al., 2008). *CASK* was found to be an essential gene for survival in mice (Atasoy et al., 2007; Srivastava et al., 2016), and consistent with that the patient population predominantly consists of females harboring a single mutant allele. *CASK* mutations in human males have been described in literature and include missense mutations exhibiting partial loss‐of‐function, mosaicism due to somatic mutation in the developing embryo, as well as more profound mutations such as deletion of exon 2 and mutation in the start codon where no protein product has been detected (Hackett et al., 2010; Kerr et al., 2019; Moog et al., 2015; Saitsu et al., 2012). Patients with undetectable CASK protein exhibit prominent cerebellar hypoplasia and electroencephalogram (EEG) defects including multifocal epileptic discharges and burst‐suppression and thus receive the diagnosis of Ohtahara syndrome (Saitsu et al., 2012). Other subjects with *CASK* null mutations that lack functional CASK protein have also been reported, and one such mutation is CASK (p.Arg27Ter) in a male patient exhibiting pontocerebellar hypoplasia, hypotonia, ventricular septal defect, optic atrophy, and intractable seizures with burst‐suppression EEG pattern (Moog et al., 2015). The CASK (p.Arg27Ter) mutation has also been described in females with severe pontocerebellar hypoplasia but no seizures were found (Hayashi et al., 2017). Notably, loss‐of‐function mutations in the *CASK* gene are reported to be associated with reduced viability in females and lethality in males either *in utero* or during infancy (Hayashi et al., 2017; Moog et al., 2015; Najm et al., 2008; Rama Devi, Lingappa, & Naushad, 2019). Here, we report a clinical case of a male patient at 17 years and 11 months of age who harbors the CASK (p.Arg27Ter) null mutation and exhibits extended survival to adolescence, raising the possibility that palliative care (i.e., an approach aimed at improving life of patients with progressive and chronic illnesses by alleviating pain and various forms of distress) (Boersma, Miyasaki, Kutner, & Kluger, 2014; Dallara & Tolchin, 2014; Oliver, 2018) could extend survival of male patients even in the absence of a functional CASK protein.

## 2 METHODS

Ethical Compliance: The Virginia Tech Institutional Review Board approved the collection and use of data from the subject. Informed consent was obtained from the family prior to participation.

The genomic DNA was extracted from peripheral blood cells and exons from *CASK* gene (NM_003688.3) were captured using the Agilent SureSelect V5 enrichment kit followed by sequencing on an Illumina HiSeq 2000, as previously described (Aldinger et al., 2019). Quantitative analysis of the EEG was done by manually binning each channel into biologically relevant frequency bands, calculating the mean power spectral density of each band, and plotting the average from each band.

## 3 CASE REPORT AND RESULTS

**Figure 1:**
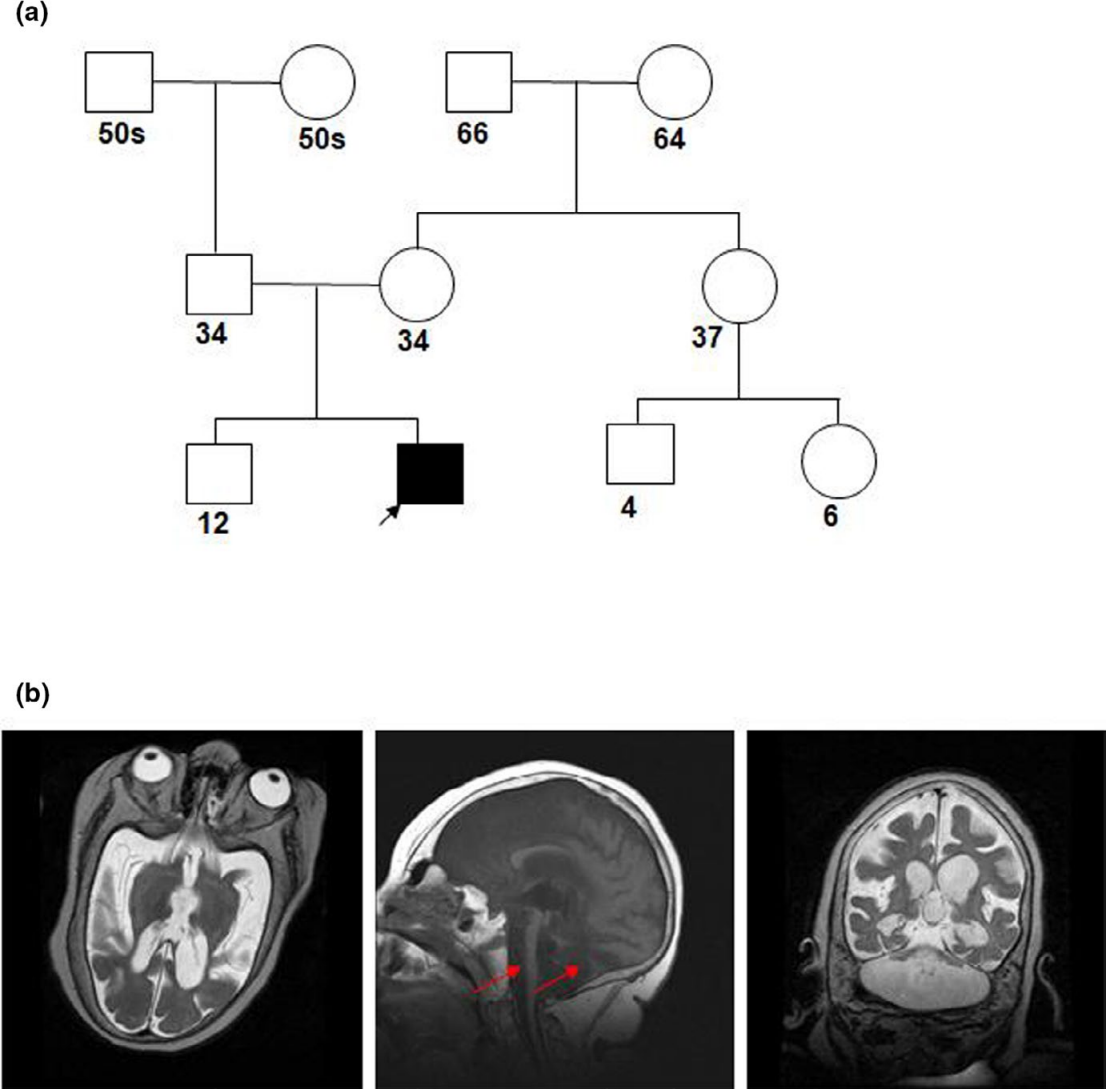
Pedigree chart and brain magnetic resonance imaging of the *CASK* R27* mutation patient. (a) Represents a three‐generation pedigree chart demonstrating the non‐inherited nature of the disorder. There is no remarkable history of the neurological disorder in the family. The numbers represent age in years and blackened square represent the proband. (b) MRI scan of the *CASK* male patient obtained at 5 years of age revealed severe hypoplasia of the cerebellum and brain stem (represented by red arrowheads) along with a supratentorial volume loss. The left, middle, and right panels depict the transverse, sagittal, and coronal planes, respectively. Footnote: *CASK* GenBank RefSeq; NM_003688.3

We report a male patient at 17 years and 11 months of age who presented with severe intellectual disability, microcephaly, and seizures. He was first evaluated at birth in the NICU (neonatal intensive care unit) due to concerns about dysmorphism and microcephaly. Neuroimaging at that time revealed severe cerebellar hypoplasia, ventriculomegaly, and brain stem atrophy/hypoplasia. A three‐generation pedigree revealed no known family history of neurological disorders or epilepsy (Figure 1a). The patient has been followed by neurology since then. Given the profound hypotonia and poor feeding, he underwent G tube placement and Nissen fundoplication prior to discharge from the NICU at 8 weeks of age. He was subsequently noted to have severe hypotonia, profound developmental delay, microcephaly, and growth retardation in early childhood. Given a history of recurrent respiratory infections and chronic aspiration, he underwent tracheostomy around age 3 and has been ventilator‐dependent since then. He is currently non‐ambulatory with severe central hypotonia and increased appendicular tone. Secondary complications have included recurrent respiratory infections requiring hospitalization, severe osteopenia with pathologic fractures, deep vein thrombosis, and autonomic instability (fluctuating body temperature, flushing, heart rate variability). Examinations of other organ systems are mostly unremarkable. Brain magnetic resonance imaging (MRI) revealed severe hypoplasia of the cerebellum and the brain stem (Figure 1b). A progressive atrophy of the supratentorial compartment is also observed, a feature that is shared by the previously described male. Magnetic resonance (MR) spectroscopy indicated a moderate decrease in n‐acetyl aspartate and an increase in choline peaks.

**Figure 2:**
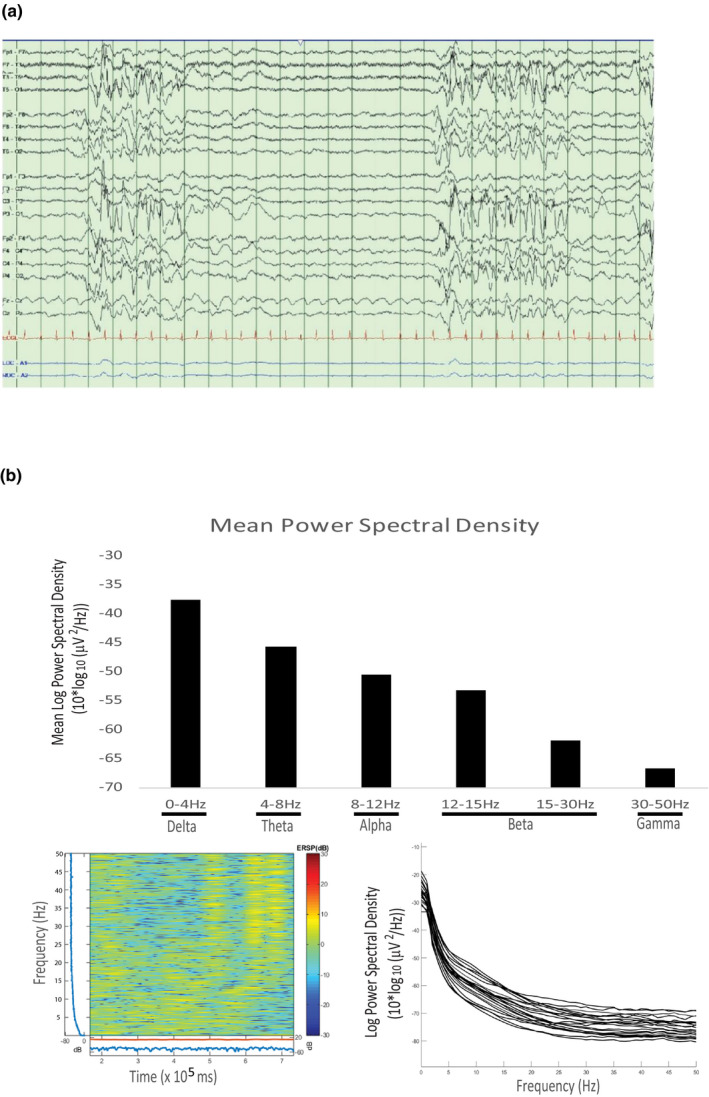
Electroencephalogram analysis of the *CASK* R27* mutation patient. (a) The EEG showing prolonged periods of attenuation and burst‐suppression along with a disorganized background and multifocal spikes. (b) Spectral analysis of the EEG at 17 years of age indicating generalized slowing. Qualitative power spectral density over 15 minutes is shown on the bottom left and the power spectral density for each channel over the entire recording is shown on the bottom right

At birth in the NICU, the patient was noted to have a severely abnormal EEG with marked disorganized background and a hypsarrhythmia pattern. Initial seizure semiology was felt to be consistent with myoclonic seizures. The EEG eventually evolved into a burst‐suppression pattern reminiscent of the infantile Ohtahara syndrome (Figure 2a). Subsequently, his seizure semiology has included tonic seizures, myoclonic seizures, and complex partial seizures. Currently, his seizures are intractable, with daily seizures despite multiple antiepileptic medications including Clobazam, Lamotrigine, Clonazepam, and Levetiracetam. He was noted to have a severe bone marrow suppression with Carbamezepine, and hyperammonemia with Valproic acid. Epileptiform activity in the form of spikes is easily observed in EEG and in 2 hours of recording a single electroclinical seizure of indeterminate onset was observed. Spectral analysis of the EEG traces confirmed an overall slowing with delta waves dominating even in the awake condition (Figure 2b).

The family underwent a diagnostic odyssey with multiple clinical diagnoses considered since birth until the trio whole exome sequencing (Trio‐WES) test recently established his molecular diagnosis. Trio‐WES analysis revealed that the patient has a *de novo CASK* (RefSeq NM_003688.3); c.79C>T (p.Arg27Ter) mutation in the second exon of the gene which introduces a nonsense mutation leading to a premature translation termination at the arginine in 27^th^ position (i.e., R27*). The R27 residue is present at the beginning of the N‐terminal canonical protein kinase domain of CASK (Mukherjee et al., 2008). Based on the genotype, it is unlikely that the brain can express any functional CASK protein from different transcript isoforms.

## 4 DISCUSSION

This case report describes for the first time an extended clinical course of a male patient harboring *CASK* R27* null mutation. Because CASK is essential for survival, it has not been possible to study the phenotypic consequences caused by constitutive *CASK* deletion in mice as it leads to neonatal death (Atasoy et al., 2007). The patient described here likely survived due to palliative care involving early tracheostomy and G‐tube insertion (Chan & Devaiah, 2009; Oliver, 2018) in the NICU, management of recurrent infections and non‐ambulatory state, as well as administration and adjustment of anti‐epileptic drugs for the management of chronic epilepsy, thus providing an opportunity to gain insights into the consequences of *CASK* null mutation beyond neonatal period up to adolescence.

Although *CASK* mutations are rare, multiple independent mutations arising at the same locus of a gene suggest that the specific locus may possess a higher susceptibility to mutation. We previously reported pathogenic genetic mutations in *CASK* that produce MICPCH (e.g., L209P and M519T), which arose independently in different individuals (LaConte et al., 2018, 2019). The CASK R27* mutation has been previously described in two girls and two boys (Hayashi et al., 2017; Moog et al., 2015; Yang et al., 2014). Intriguingly, we are aware of a 2‐year‐old boy with R28* mutation exhibiting infantile spasm, dystonia, and developmental delay. The R28* mutation was also previously reported in a girl with intellectual disability, pontine, and cerebellar atrophy who developed spasms at 3 years of age, but the seizures were controlled with antiepileptic drugs (Michaud et al., 2014). Finally, the variant R28L has been described in a male patient presenting with FG syndrome (Piluso et al., 2009). Thus, the clustering of mutations at these two arginine residues (i.e., R27 and R28) at the beginning of the N‐terminal kinase domain of CASK suggests that this region may constitute a mutational hotspot. In fact, the arginine codon “CGA” is known to be a hotspot for conversion into a stop codon due to a C‐to‐T transition (Cooper, Mort, Stenson, Ball, & Chuzhanova, 2010; Cowell, Smith, & Bia, 1994). Premature stop codons not only truncate the protein but also activate machinery for the nonsense‐mediated decay of RNA (Romanov & Sukhoverov, 2017).

Although haploinsufficiency of *CASK* gene in females might present with seizures with incomplete penetrance (~40%) (Moog, Uyanik, & Kutsche, 1993), hemizygous *CASK* mutations in males produces catastrophic neurodevastation and epileptic encephalopathy (Moog et al., 2015; Saitsu et al., 2012). *CASK* is an X‐linked gene and is thus subject to random inactivation in females. *CASK* mutations, therefore, generate mosaicism in female brains, with only 50% of cells expressing the mutant version of CASK; this may explain the diminished severity of phenotypes in girls. In fact, both of the girls reported to have the heterozygous CASK R27* mutation exhibited microcephaly, pontocerebellar hypoplasia, and profound intellectual disability, but no seizures (Hayashi et al., 2017). Surprisingly, in murine models, no secondary selection of CASK‐positive cells has been noted, and to some extent, the function of CASK seems to be non‐cell autonomous (Kerr et al., 2019; Srivastava et al., 2016).

The subject described here, a male with CASK R27* null mutation, shares similarities with the previously reported case (Moog et al., 2015) in having short stature, seizures with EEG abnormalities, severe pontocerebellar hypoplasia, and microcephaly. Although the previous case manifested with a relevant ventricular septal defect (VSD) and cardiac insufficiency, cardiac functioning is normal in our patient. Strikingly, in both reported cases of CASK R27* mutation in males, a progressive cerebral atrophy has been noted (Moog et al., 2015; Yang et al., 2014). The precise mechanisms by which CASK loss produces a profound neurodevelopmental/degenerative defect or seizures remain uncertain. It has been suggested that CASK may act *via* its interaction with Tbr1 (Hsueh, Wang, Yang, & Sheng, 2000), but in a murine model, disruption of the CASK‐Tbr1 interaction did not produce any structural defects or epilepsy (Huang & Hsueh, 2016). CASK is also a presynaptic scaffolding molecule (Butz, Okamoto, & Sudhof, 1998; Mukherjee et al., 2008), and thus lack of CASK could produce synaptic defects. However, in a murine model of constitutive *CASK* deletion, it was reported that CASK loss did not affect the core neuronal functions of excitability, calcium‐dependent presynaptic release, or postsynaptic receptor arrangement (Atasoy et al., 2007). The only changes observed in the constitutive *CASK* deletion murine model were in action potential‐independent spontaneous release (Atasoy et al., 2007). It is unlikely that a minor electrophysiological change alone would manifest as such a profound neurodevelopmental condition. Recent work suggests that CASK may play critical roles in regulating mitochondrial, cytoskeletal, and protein metabolic functions (Mukherjee, Slawson, Christmann, & Griffith, 2014; Patel et al., 2020; Srivastava et al., 2016), but how these diverse functions of CASK relate to the observed disorder remains to be investigated.

Finally, boys with *CASK* mutations may present with Ohtahara syndrome, a type of epileptic encephalopathy displaying a burst‐suppression EEG pattern (Moog et al., 2015; Saitsu et al., 2012). Epileptic encephalopathies are a heterogeneous group of epileptic disorders accompanied by a progressive encephalopathy (Khan & Al Baradie, 2012). It has been argued that refractory seizures in epileptic encephalopathies may contribute to this progression (Howell, Harvey, & Archer, 2016). The patient described here exhibited infantile spasms, which are also a type of epileptic encephalopathy that is often accompanied by a hypsarrhythmia pattern in the interictal EEG. Over time, the patient's EEG pattern evolved to display a burst‐suppression pattern mimicking Ohtahara syndrome and subsequently evolved into tonic and myoclonic seizures. It is possible that with a genetic etiology of epileptic encephalopathy such as *CASK* mutations, the epileptic seizures may be a part of progressive encephalopathy, rather than a primary event, and hence evolve over time (encephalopathy with epilepsy) (Helbig, von Deimling, & Marsh, 2017; Khan & Al Baradie, 2012). Notably, a more general analysis of the patient's EEG at the age of 17 years indicated an overall slowing, as well as pronounced attenuation.

In conclusion, with early clinical management and palliative interventions that assist with respiration and nutrient delivery, subjects born without functional CASK protein may develop beyond infancy, albeit with profound neurodevelopmental disability. Future studies are warranted to examine the underlying pathogenic mechanisms and associated diverse functions of CASK.

## CONFLICT OF INTEREST

The authors declare no potential conflict of interest.

## AUTHOR CONTRIBUTIONS

KM and SS conceptualized the study. PAP and DSR analyzed clinical findings and generated figures. KM, DSR, LEWL, and SS interpreted the findings and wrote the manuscript. KM and SS performed manuscript revision. All authors approved the final version of the manuscript.
